# Prolonged response to combined BRAF and MEK inhibition in BRAF mutant colorectal cancer, a case report

**DOI:** 10.3389/fonc.2026.1829831

**Published:** 2026-07-16

**Authors:** Michela Guardascione, Monica Rizzetto, Luisa Foltran, Paola Di Nardo, Marco de Scordilli, Elena Ongaro, Arianna Fumagalli, Anna Calabrò, Andrea Botticelli, Paolo Marchetti, Fabio Puglisi

**Affiliations:** 1 Department of Medical Oncology, Centro di Riferimento Oncologico di Aviano (CRO), Istituto di Ricovero e Cura a Carattere Scientifico (IRCCS), Aviano, Italy; 2 Department of Medicine, University of Udine, Udine, Italy; 3 Medical Oncology Unit, Tor Vergata University Hospital, Rome, Italy; 4 Department of Nuclear Medicine, Centro di Riferimento Oncologico di Aviano (CRO), IRCCS, Aviano, Italy; 5 Department of Radiological, Oncological and Pathological Sciences, Sapienza University of Rome, Rome, Italy; 6 Oncology Unit, Azienda Ospedaliero-Universitaria (AOU) Policlinico Umberto I, Rome, Italy; 7 Department of Oncology, Istituto Dermopatico dell'Immacolata - Istituto di Ricovero e Cura a Carattere Scientifico (IDI - IRCCS), Rome, Italy

**Keywords:** BRAF mutation, case report, colorectal cancer, MEK inhibitor, resistance

## Abstract

**Background:**

BRAF V600E-mutant metastatic colorectal cancer (BRAF mCRC) is often associated with limited durable responses. After the publication of the randomized phase III BEACON trial investigating chemo-free regimens including BRAF inhibitors, anti-EGFR drugs, and MEK inhibitors (MEKis) in pre-treated BRAF mCRC patients, no MEKis have entered into clinical practice so far. Moreover, the recent phase III BREAKWATER trial investigating the first-line treatment of BRAF mCRC patients did not include MEKis at all. We present a compelling clinical case of a patient with BRAF mCRC who has experienced an extraordinary response to his MEKi-based treatment.

**Case presentation:**

In 2019 a 60 years old male patient was diagnosed with BRAF mCRC (peritoneal metastases). The patient was asymptomatic, with PS 0. He was started on first-line treatment with FOLFOXIRI plus bevacizumab and after 8 cycles underwent curative surgery. One year later, due to peritoneal relapse, second-line treatment with the BRAF inhibitor vermurafenib combined to the MEKi cobimetinib was started within a phase II clinical trial. The patient has maintained stable disease with manageable side effects over nearly four years. He is still alive and in good clinical conditions.

**Conclusion:**

The prolonged progression-free survival seen in the present clinical case represents, to our knowledge, the longest documented response to a MEKi-based regimen in this setting, supporting the hypothesis that MEKis deserve reconsideration in mCRC treatment. This case highlights the value of personalized genomic profiling and practical clinical decision-making, offering insights into treatment sequencing, resistance mechanisms, and future targeted therapy strategies in BRAF mCRC.

## Introduction

Approximately 10% of metastatic CRC (mCRC) patients (pts) harbor a BRAF mutation, mostly V600E, which is linked to poor prognosis (1) and reduced benefit from standard chemotherapy (2). Targeted therapy including BRAF inhibitors (BRAFi) plays a crucial role in this subtype of mCRC pts even though BRAFi are not effective as single agents (3). In this scenario MEK inhibitors (MEKi) may contribute to block downstream signaling and anti-EGFR drugs (aEGFR) might prevent feedback activation. The combination of BRAFi (e.g., dabrafenib, encorafenib, or vemurafenib) with MEKi (e.g., trametinib, binimetinib, or cobimetinib) has been investigated. In particular, the phase II basket TAPUR trial tested vemurafenib plus cobimetinib in a population of pretreated BRAF V600E mutated mCRC (BRAF mCRC) pts, showing a disease control rate (DCR) of 52% and an overall response rate (ORR) of 30%; grade ≥ 3 (according to the CTCAE grading system) adverse events (AEs) were reported in 43% of pts (4). Moreover, the combination of BRAFi and aEGFR (e.g., cetuximab, panitumumab) has shown clinical efficacy. The randomized phase III BEACON study enrolled 665 previously treated BRAF mCRC pts who were randomly assigned to one of three arms: encorafenib-binimetinib-cetuximab (triplet), encorafenib-cetuximab (doublet), or cetuximab-irinotecan/FOLFIRI (control). The updated analysis showed a median overall survival (mOS) of 9.3 months for both the triplet and the doublet groups, and 5.9 months for the control. ORRs were 26.8% for the triplet, 19.5% for the doublet, and 1.8% for the control. Grade ≥3 AEs were 58% in the triplet and 50% in the doublet groups (5).

Here we present a case report on a patient with BRAF mCRC, who exhibited an exceptional response to vemurafenib plus cobimetinib lasting for over three years.

## Case presentation

**Figure 1:**
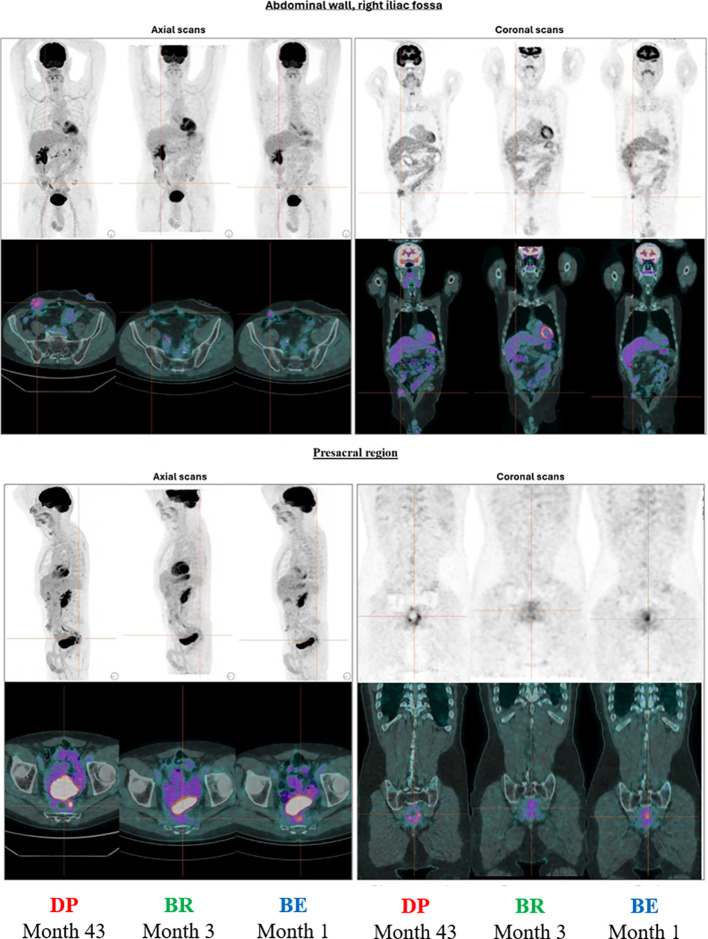
PET scan images with pathological nodules in the abdominal wall (at the level of the right iliac fossa) and in the presacral region: Basal Evaluation (BE), Best Response (BR) and Disease progression (DP).

In 2016, due to the evidence of mild to moderate anemia, a 57 years old male patient underwent a colonscopy, showing a right-sided colon cancer. Right hemicolectomy was performed, with pathology reporting a pT4 N0 (0/19) adenocarcinoma; molecular characterization demonstrated proficient MMR status, wild type KRAS/NRAS, and V600E mutation of BRAF. This patient had neither family history nor relevant comorbidities, apart from benign prostatic hypertrophy and hypertension for which he was taking, respectively, tamsulosin and ramipril. An adjuvant treatment with capecitabine was administered. In 2019 the patient received a new diagnosis of adenocarcinoma of the rectosigmoid junction; hence, he underwent a neoadjuvant chemo-radiotherapy followed by Hartmann’s resection surgery. At surgical examination a suspicious right diaphragmatic peritoneal nodule was identified and thus biopsied. The pathology report showed a ypT2 N1c (a tumor deposit among 6 resected lymph nodes) adenocarcinoma; the peritoneal lesion resulted in metastasis from adenocarcinoma. The molecular profile showed a BRAF V600E mutation. At radiological re-staging, pelvic peritoneal and local relapse/residual disease were found, therefore the patient was started on first-line treatment with FOLFOXIRI plus bevacizumab; 8 cycles were administered, achieving a partial response. After multidisciplinary discussion, Miles’ surgery, peritoneal debulking, intra-operative radiotherapy and HIPEC were performed in September 2020, with no residual pathologic disease; BRAF V600E mutation was confirmed. In September 2021, due to an increase of the tumor markers in the presence of a negative CT scan, a 18F-FDG PET/CT scan was performed showing high-metabolism of one nodule in the abdominal wall and another in the pre-sacral region, associated with left hydroureteronephrosis. The disease recurrence was histologically confirmed; a nephrostomy was advised, but the patient refused to undergo the procedure. At this point he was offered either the standard second-line treatment with encorafenib plus cetuximab or the enrollment in a clinical study; in accordance with his preferences, after signing the informed consent form, he entered a phase II randomized clinical trial (ROME) investigating the efficacy of target therapy according to tumor genomic evaluation regardless of histology (6). A baseline 18F-FDG PET/CT scan was repeated in November 2021 (**Figure 1**).

**Figure 2:**
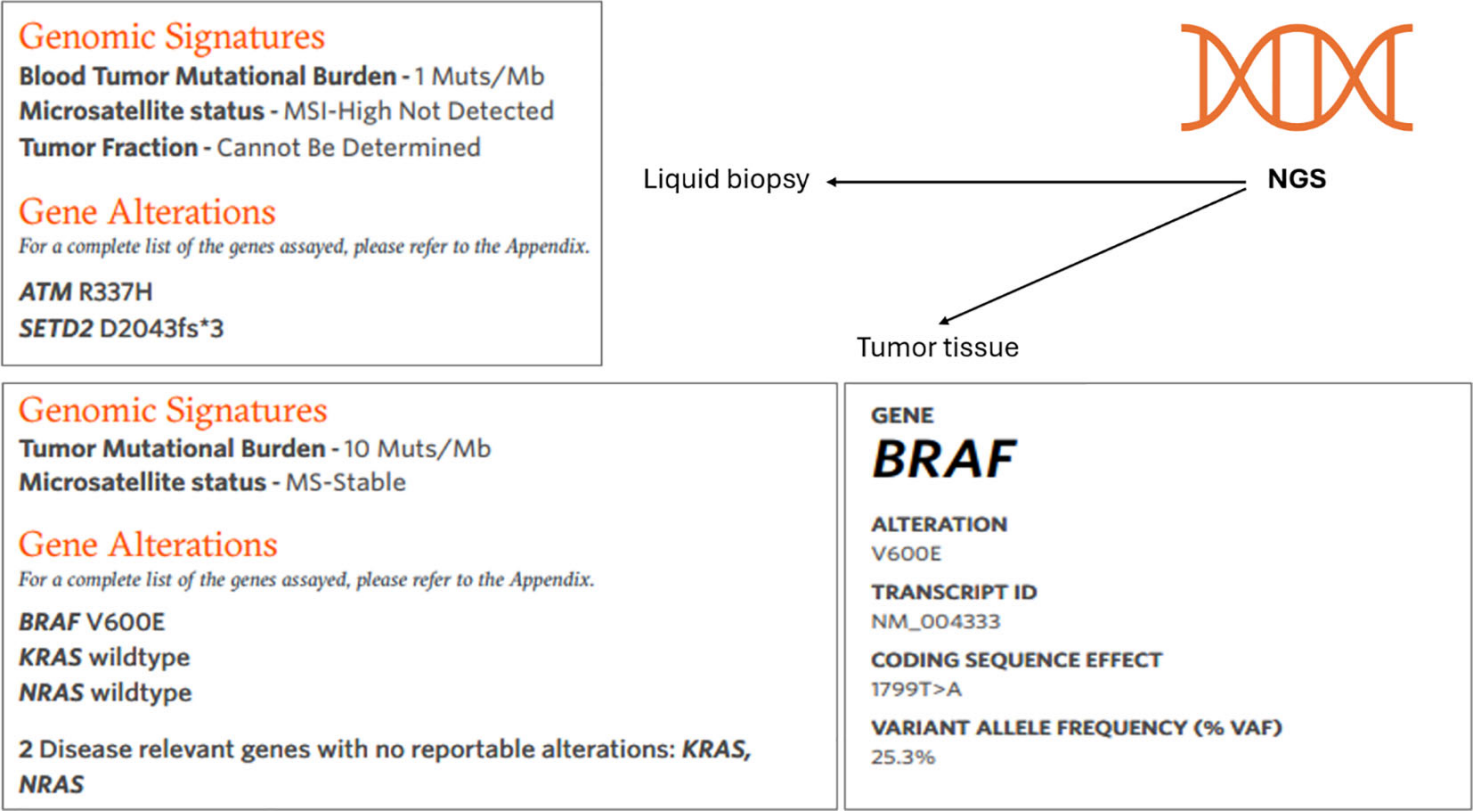
NGS on liquid biopsy and tumor tissue.

A Next Generation Sequencing (NGS) of the tumor tissue (*Foundation One* ^®^ CDx assay with 324 gene panel) confirmed BRAF V600E mutation with variant allele frequency (VAF) of 25.3%; Tumor Mutational Burden (TMB) was 10 Muts/Mb, with microsatellite status being stable. NGS on liquid biopsy did not identify BRAF mutation, however the negative result of Tumor Fraction, reported as *cannot be determined*, can explain the discordance between tumor tissue and liquid biopsy analyses (**Figure 2**).

**Figure 3:**
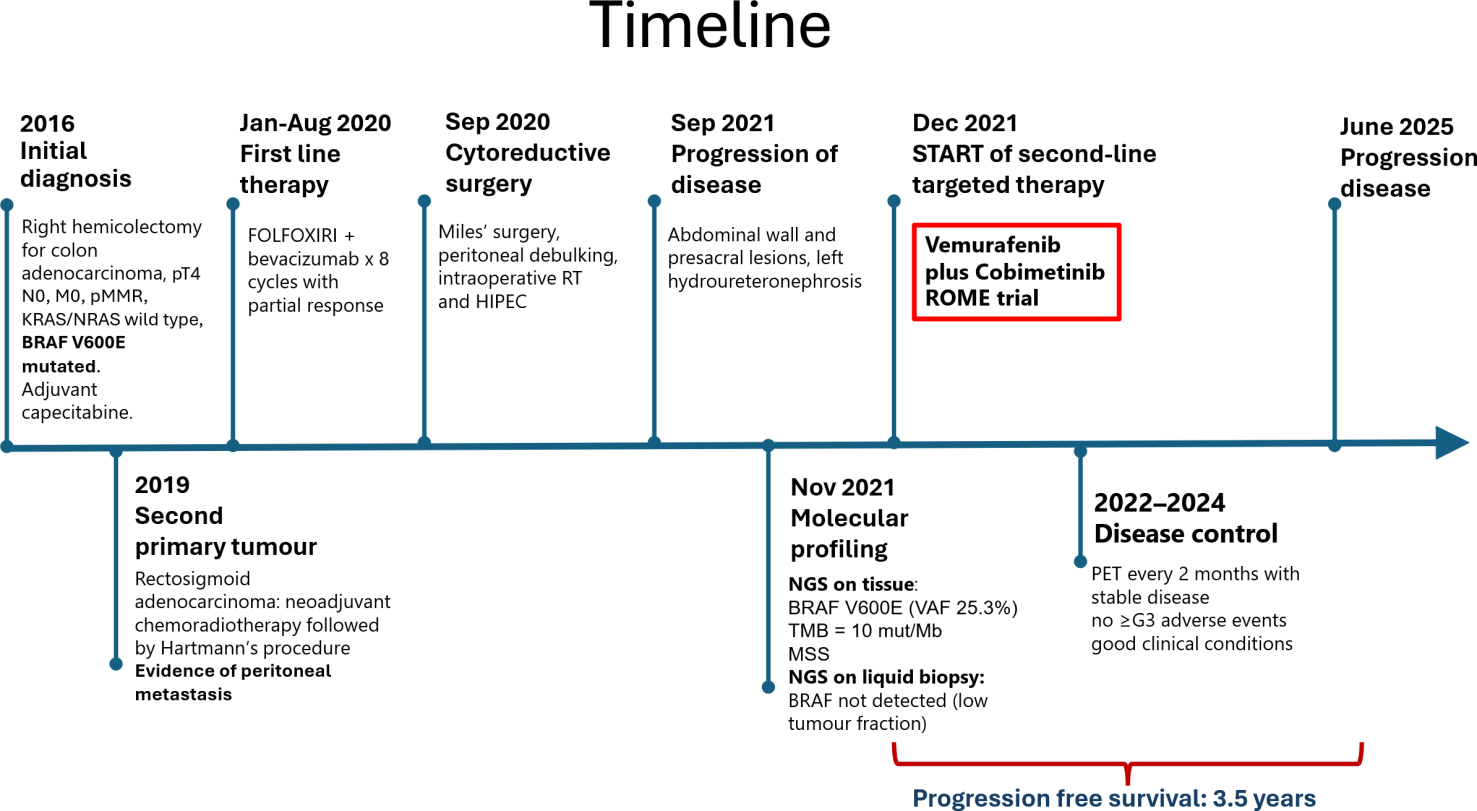
Timeline.

In December 2021 the patient was enrolled into the experimental study arm and started on vemurafenib plus cobimetinib. Vemurafenib was administered at a personalized starting dose of 720 mg (versus standard dose of 920 mg) twice a day, considering the mild hypercreatininemia at baseline, while cobimetinib was prescribed at standard dose of 60 mg once a day. The patient has experienced no grade ≥ 3 AE. As per study protocol, disease re-evaluation has been performed by 18F-FDG PET/CT scan every 2 months, showing stable disease as best response to treatment by PERCIST criteria although with a trend in favor of metabolic and dimensional reduction (**Figure 1**). In June 2025 the 18F-FDG PET/CT scan showed an increased hyper uptake of the nodule in the abdominal wall for which, after multidisciplinary discussion, in August 2025 the patient underwent laparotomy aiming at removing the only progressing lesion and potentially re-starting the same systemic treatment the patient has been on so far. In this regard, a liquid biopsy outside of the study protocol has recently been performed showing the presence of BRAF V600E mutation. Currently the patient has not got yet the chance to re-start the oncological treatment due to the worsening of left hydroureteronephrosis even though his performance status remains good. The **Figure 3** shows the timeline of events in this clinical case.

## Discussion

This clinical case, although representing a single experience, supports the use of MEKi in treating BRAF mCRC pts and emphasizes the convenience of an all-oral regimen. Clinical trials with MEKi in the setting of mCRC followed a different course in comparison to advanced melanoma, a context in which four randomized phase III trials with BRAFi plus MEKi showed the superiority of doublets compared to BRAFi monotherapy. Conversely, no direct comparison testing the combination of MEKi plus BRAFi versus single agent BRAFi has been available so far in mCRC, and more importantly there is lack of data on directly comparing BRAFi plus MEKi to BRAFi plus aEGFR as well as each doublets versus triplet with BRAFi, MEKi, and aEGFR. Although EGFR plays a critical role in both primary and acquired resistance to BRAFi in BRAF mCRC, other mechanisms may explain some cases of acquired resistance, such as alterations in the MAPK pathway components including MEK mutations.

BEACON (5) was the first phase III trial conducted in BRAF mCRC pts and played a central role in changing the clinical practice in this subgroup of pts. Based on the results from this trial, in June 2020 encorafenib was FDA-approved in combination with cetuximab for previously treated pts. Initially, the submission also included binimetinib, although during the procedure the drug was withdrawn due to the lack of survival advantage reported in the study. However, the BEACON trial was not powered to compare doublet vs triplet as well as did not test dual inhibition of BRAFi plus MEKi; therefore the real benefit of MEKi is difficult to estimate based on the results from this study. Considering the study population of previously treated BRAF mCRC pts, the ORR of 26.8% observed for the triplet, compared to 19.5% for the doublet, is not negligible. Moreover, although the rate of AEs ≥ grade 3 was higher for triplet than doublet (65.8% vs 57.4%), the discontinuation rate was the same (9%) in both triplet and doublet arms. Some AE including headache, musculoskeletal pain, arthralgia, and myalgia occurred more frequently with the doublet than the triplet, which is consistent with the toxicity mitigation of BRAF inhibition induced by MEKi (7). In addition, as shown by Corcoran et al. in a clinical study involving 43 pts with heavily pretreated BRAF mCRC, MAPK suppression in on-treatment tumor biopsies occurred only in a subgroup of BRAF mCRC pts treated with dabrafenib plus panitumumab, suggesting a potential role for an EGFR-independent MAPK reactivation (8). Collectively, these data underscore the need to block both EGFR-dependent and -independent pathways in BRAF mCRC. Disappointingly, nearly two years after the approval of encorafenib plus cetuximab doublet in previously treated BRAF mCRC pts, the combination of dabrafenib and trametinib was FDA-approved as tissue-agnostic therapy for all previously treated solid tumors harboring BRAF V600E mutations, though excluding mCRC pts. All these regulatory steps, despite being great advances in medical oncology, emphasize the importance of repurposing MEKi in the context of mCRC.

Following the BEACON trial, a single-arm phase II study of first-line triplet with encorafenib plus cetuximab plus binimetinib in BRAF mCRC showed that a chemotherapy-free option was active and feasible. The mOS of 18.3 months reported in this study is among the longest survival seen in BRAF mCRC pts (9). Currently, the global phase III BREAKWATER trial is investigating chemotherapy plus encorafenib and cetuximab vs standard of care as first-line treatment for patients with BRAF mCRC pts. The experimental treatment has recently showed significant improvement in ORR; at the second interim analysis (presented at ASCO Congress 2025) the hazard ratios for progression free survival and overall survival were 0.53 (95% CI: 0.407-0.677) and 0.49 (95% CI: 0.375-0.632) respectively (10). This trial is not investigating the addition of MEKi, though.

The NGS performed on tumor tissue from the present clinical case showed relatively high VAF of BRAF mutation and TMB. Interestingly, exploratory analyses on circulating tumor DNA from a small cohort of pts from the BEACON study found that pts with high BRAF mutant allele fraction could derive greater benefit from the addition of MEKi (11). Moreover, in a multicenter case-control study a higher TMB was associated with limited benefit from BRAFi plus aEGFR in pts with BRAF mCRC (12).

Interestingly, our clinical case reinforces the utility as well as the feasibility of performing disease re-evaluation by 18F-FDG PET/CT scan. Indeed, this exam has been much helpful during all the clinical course of the patient, providing clinicians with reliable parameters of therapeutic response. In conclusion, the present clinical report describes a remarkable long-term response to MEKi-based treatment in a patient with BRAF mCRC, marking the first documented case of such prolonged PFS with this unfavorable mutation. This excellent outcome, along with the scientific evidence, draws the attention to the role of MEKi in this subgroup of mCRC pts. Ongoing studies combining BRAFi and MEKi with ERK inhibition or immune checkpoint inhibitors could be a promising strategy in the next future (i.e. NCT05308446; NCT04294160).

## Patient perspective

The patient has been deeply involved into the decision making process from the very beginning. Indeed, he clearly expressed how confident he felt with starting a treatment in the context of a clinical trial rather than receiving standard treatment. This attitude of the patient was crucial for us in order to abandon a potentially effective treatment (encorafenib plus cetuximab) in favor of an experimental one. The patient maintained a very good compliance throughout the treatment with a totally acceptable tolerance, as he himself has said several times.

## Data Availability

Individual patient data generated during the ROME trial are available upon reasonable request from academic or qualified clinical researchers affiliated with recognized institutions, strictly for the purpose of conducting non-commercial, ethically approvable research aligned with the original scope of the trial. The trial registration, study protocol and methodological details are publicly accessible through ClinicalTrials.gov (accession identifier NCT04591431; https://clinicaltrials.gov/ct2/show/NCT04591431). Additional publicly available datasets used in the analysis include the ClinVar database: freely accessible at https://ww.ncbi.nlm.nih.gov/clinvar/; the OncoKB database: accessible at https://www.oncokb.org/; the COSMIC database: accessible at https://cancer.sanger.ac.uk/cosmic; and the ESMO ESCAT scale: accessible at https://www.esmo.org/guidelines/esmo-scale-for-clinical-actionability-of-molecular-targets-escat. No other public repositories or datasets requiring accession codes were used in this study.
